# Disagreement on expectations: firms versus consumers

**DOI:** 10.1007/s43546-021-00164-4

**Published:** 2022-01-10

**Authors:** Oscar Claveria

**Affiliations:** Department of Econometrics, Statistics and Applied Economics, AQR-IREA, University of Barcelona, Diagonal, 690, 08034 Barcelona, Spain

**Keywords:** Economic uncertainty, Geometry, Expectations, Disagreement, Business and consumer surveys, Vector autoregressions, C13, C32, C51, D84, E23

## Abstract

This paper evaluates the dynamic response of economic activity to shocks in agents’ perception of uncertainty. The study focuses on the comparison between manufacturers’ and consumers’ perception of economic uncertainty, gauged by a geometric discrepancy indicator to quantify the proportion of disagreement in eleven European countries and the Euro Area. A vector autoregressive framework is used to estimate the impulse response functions to innovations in disagreement, both for manufacturers and consumers. The effect on economic activity of shocks to the perception of uncertainty is found to differ markedly between both types of agents. On the one hand, shocks to consumer discrepancy tend to be of greater magnitude and duration than those to manufacturer discrepancy. On the other hand, innovations in disagreement between the two collectives have an opposite effect on economic activity: shocks to manufacturer discrepancy lead to a decrease in economic activity, as opposed to shocks to consumer discrepancy. This finding is of particular relevance to researchers when using cross-sectional dispersion of survey-based expectations for approximating and assessing economic uncertainty, since the effect on economic growth of shocks to disagreement may be dependent on the type of agent and the way in which expectations have been elicited.

**Supplementary Information:**

The online version contains supplementary material available at 10.1007/s43546-021-00164-4.

## Introduction

The analysis of economic uncertainty has gained renewed interest since the advent of the 2008 financial crisis. While there is a widespread consensus that uncertainty shocks have an effect on real activity (Bachmann and Bayer 2013; Baker et al. 2016; Bloom 2009; Paloviita and Viren 2014), the question of what exactly is meant by economic uncertainty and how to measure it are aspects that are still open to debate (Dibiasi and Sarferaz 2020; Rossi et al. 2020). In order to provide insight into the nature of the shocks that drive business cycles, Kozeniauskas et al. (2018) differentiated between three types of uncertainty—micro uncertainty (cross-sectional variance of firm-level outcomes), macro uncertainty (aggregate shocks) and higher-order uncertainty (disagreement), showing that the three measures are statistically distinct. Glas (2020) and Rich and Tracy (2021) also delved into the relationship between macroeconomic uncertainty and forecaster disagreement, arriving to similar conclusions. Specifically, Glas (2020) found that survey-based uncertainty is associated with overall policy uncertainty, while disagreement is related more closely to the expected fluctuations on financial markets. Other studies that analyse the linkage between uncertainty and disagreement are those of Krüger and Nolte (2016) and Lahiri and Sheng (2010).

A reflection of the difficulty of specifying what exactly is understood by uncertainty shocks and disentangling them from other type of shocks, are the different strategies used to measure uncertainty. In a recent paper, Binge and Boshoff (2020) grouped the different approaches to proxy economic uncertainty into five categories: those based on financial data, text-based proxies, econometrically constructed measures, disagreement among professional forecasters, and responses from business and consumer surveys.

Examples of the first approach vary depending on the type of variable (bond yields, exchange rates, etc.). Some authors have opted to proxy it using the realized volatility in equity markets (Basu and Bundick 2017; Bekaert et al. 2013; Caggiano et al. 2017; Yıldırım-Karaman 2017), while others in oil prices (Hailemariam et al. 2019) or in the prices of natural gas (Atalla et al. 2016; Hailemariam and Smyth 2019). In a recent paper, Caggiano and Castelnuovo (2021) combined volatility data on stock market, exchange rate returns and bond yields, to construct a measure of global uncertainty.

Since developments of the stock market only partially reflect developments of the real economy (Girardi and Reuter 2017), some authors collect new data for approximating economic uncertainty. The most popular approach is based on calculating the frequency with which concepts related to uncertainty appear in the media. Baker et al. (2016) constructed the economic policy uncertainty (EPU) index by combining a text-mining measure with disagreement among forecasters together with the number of tax code previous about to expire. With this aim, Aromi (2020) used word vector representations. To bypass the fact that this approach is limited by the degree of subjectivity entailed in the selection of newspapers and search terms, Altig et al. (2020) recently used an alternative measure based on Twitter chatter about economic uncertainty.

A third way to proxy uncertainty is by means of model-based measures. Jurado et al. (2015) proposed using econometric unpredictability, understood as the conditional volatility of the unforecastable components of a broad set of economic variables. This methodology, based on the aggregation of the variance of forecast errors, has been used inter alia by Chuliá et al. (2017) and Meinen and Roehe (2017). The ex-post nature of this approach has recently generated a strand of the empirical research that makes use of more direct measures of uncertainty based on the information elicited from surveys (Binder 2017; Binding and Dibiasi 2017; Mitchell et al. 2007; Mokinski et al. 2015).

Survey-based measures of economic uncertainty are usually obtained through different dispersion metrics computed from forecast surveys. The ex-ante nature of these surveys makes them especially appropriate to evaluate the anticipatory properties of uncertainty shocks. Some recent works that take advantage of this type of information are, for example, those of Rich and Tracy (2021) and Rossi and Sekhposyan (2017) for the Euro Area (EA), and that of Jo and Sekkel (2019) for the US. Altig et al. (2021) carried out their own survey, the monthly panel Survey of Business Uncertainty, to construct monthly indices of business expectations (first moment) and uncertainty (second moment) for the US private sector. For an overview of recent developments regarding the measurement of uncertainty, see Castelnuovo (2019) and Cascaldi-Garcia et al. (2020).

Dispersion-based proxies of economic uncertainty vary depending on the type of survey information they are based on. Surveys such as the Survey of Professional Forecasters (SPF) conducted quarterly by the Philadelphia Fed ask respondents to give point forecasts and to attach a probability to each of a number of pre-assigned intervals over which their forecast may fall. Consequently, the SPF has been widely used to derive and assess different proxies of economic uncertainty (Clements and Galvão 2017; Dovern 2015; Krüger and Nolte 2016; Mankiw et al. 2004; Oinonen and Paloviita 2017; Rich and Tracy 2009; Rossi and Sekhposyan 2015; Rossi et al. 2020). While providing researchers with both point and density forecasts for the US, the SPF is based on a limited sample of forecasters (Sill 2012).

Other surveys, such as the business and consumer surveys (BCS) conducted by the European Commission, ask respondents about the expected direction of change of a wide range of economic variables. These surveys, in addition to being publicly available, have the advantage of being carried out monthly in a large number of European states, allowing comparability between countries. Since the results of BCS are qualitative in nature, Bachmann et al. (2013) proposed an uncertainty measure based on the disagreement in production expectations.

Since then, these types of surveys have been used frequently to obtain alternative measures of disagreement and to evaluate their effects on different macroeconomic variables. Using aggregate BCS data instead of micro data, Girardi and Reuter (2017) presented three survey-based uncertainty indicators. Aggregate BCS data have also been used to assess the impact of uncertainty about growth, inflation and employment on their corresponding macro aggregates (Claveria 2021a), as well as to evaluate the impact on economic activity of adding different dimensions of disagreement among agents (Claveria 2021b). In a recent paper, Glocker and Hölzl (2021) presented a direct measure of economic uncertainty based on a business survey for the Austrian economy in which firms are asked directly about their degree of certainty related to their business situation.

Given that BCS incorporate a non-response option (‘don’t know’), Dibiasi and Iselin (2019) proposed using the share of responses in forward-looking questions to directly approximate Knightian uncertainty. This approach allows to capture the proportion of firms that do not formalise expectations about their future demand. Using firm-level data for Germany, Bachmann et al. (2020) found evidence that Knightian responses are indeed motivated by a lack of clarity about the future, and that firms report more subjective uncertainty after either high or low growth realizations (Bachmann et al. 2018). Due to their reliance on firm-level information, this type of indicators become frequently associated with idiosyncratic (micro)uncertainty (Bloom 2014).

Therefore, while the debate regarding the appropriate way to measure an unobservable phenomenon such as uncertainty is still open, there is a general consensus regarding the effect that uncertainty shocks have on economic activity (Caggiano et al. 2017; Caggiano and Castelnuovo 2021; Carriero et al. 2018; Netšunajev and Glass 2017). In this sense, the seminal works of Baker et al. (2016) and Bloom (2009) showed that economic uncertainty has a negative impact on economic growth. Angeletos et al. (2021) recently found that the behaviour of expectations about other variables, such as inflation and unemployment, may be totally different of that of economic activity. Additionally, the economic theory identifies a number of channels through which uncertainty can alter the decisions of private agents, namely, firms and households (Basile and Girardi 2018). Since BCS allow the calculation of measures of disagreement for different types of agents, whether they are companies from different economic sectors, or different types of consumers, this paper compares the effects of shocks on uncertainty both from the perspective of demand and supply. To this end, based on the geometric indicator of discrepancy proposed by Claveria et al. (2019), the level of disagreement among firms (DB) and among consumers (DC) is computed for each country in the sample.

In business surveys, firms are asked about expected production, selling prices, employment and other variables concerning developments in their sector, while households are asked about their spending intentions and the expected general economic situation influencing those decisions. We use information coming from both surveys to elicit agents’ expectations about production and economic activity in eleven European countries and the EA: Austria (AT), Belgium (BE), Finland (FI), France (FR), Germany (DE), Greece (EL), Italy (IT), the Netherlands (NL), Portugal (PT), Spain (ES), and the United Kingdom (UK).

By disentangling between the level of disagreement between manufacturers regarding production expectations (supply side) from that among consumers regarding expectations about the economic situation (demand side), the dynamic response of economic growth to innovations in each type of disagreement (manufacturers’ vs consumers’) is analysed by means of a vector autoregressive (BVAR) framework. This study contributes to the existing literature by providing a cross-country comparative of the dynamic relationship between the perception of economic uncertainty and the evolution of economic activity from both the supply and the demand sides of the economy.

The paper is organised as follows. The next section introduces the data and the methodological approach used to measure disagreement. Empirical results are provided in Sect. 3. Finally, concluding remarks and future lines of research are drawn.

## Data and methodology

### Data

This section describes the survey data that are used to compute disagreement. The empirical analysis focuses on manufacturing firms’ and consumers’ expectations about the future evolution of economic activity, which are taken from the joint harmonised EU industry and consumer surveys conducted monthly by the European Commission. Economic activity is approximated by the growth rate of the gross domestic product (GDP) provided by Eurostat. To deal with the different frequencies of the data set, temporally disaggregated quarterly GDP data are obtained by linear interpolation. The sample period goes from January 2005 to December 2019.

In the survey, manufacturers are asked about their expectations regarding production, selling prices and employment for the months ahead, and they are faced with three options: “up”, “unchanged” and “down”. The aggregated percentages of the individual replies in each category are, respectively, denoted as *P*, *E*, and *M*.

Consumers, for their part, are asked how they think the general economic situation, the cost of living, and the level of unemployment in the country will change over the next 12 months. Consumers have three additional response categories: two at each end (“a lot better/much higher/sharp increase”, and “a lot worse/much lower/sharp decrease”), and a “don’t know” option. We opt for grouping all positive responses in *P*, all negative ones in *M*, and incorporating the “don’t know” share in *E* for each time period.

### Measurement of uncertainty

The most common way of presenting survey results is the balance, obtained as $P_{t} - M_{t}$. The most widespread measures of disagreement among survey respondents use the dispersion of balances as a proxy for uncertainty (Bachmann et al. 2013; Girardi and Reuter 2017). Bachmann et al. (2013) proposed an indicator of disagreement based on the square root of the variance of the balance:

1 $${\text{DISP}}_{t} = \sqrt {P_{t} + M_{t} - (P_{t} - M_{t} )^{2} }.$$

**Fig. 1:**
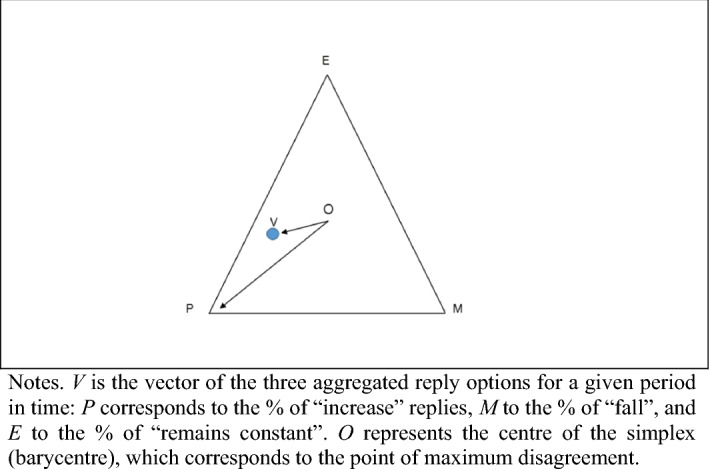
Projection of the combination of the three reply options

The omission of the information contained in the “no change” category led Claveria et al. (2019) to develop a disagreement metric that incorporated the information coming from all the reply options, whose number is denoted as *N*. Given that the sum of the shares of responses adds to a hundred, the authors compute an *N*-dimensional vector that aggregates the information from all answering categories and project it as a point on a simplex of $N - 1$ dimensions that encompasses all possible combinations of responses. For $N = 3$, the simplex takes the form of an equilateral triangle (Fig. 1), where the point $V$ corresponds to a unique convex combination of the three reply options for each period in time. See Claveria (2019) for an application of the methodology when $N = 5$.

Insomuch as all vertices are at the same distance to the centre of the simplex ($O$), the ratio of the distance of a point to the barycentre ($VO$) and the distance from the barycentre to the nearest vertex ($OP$) provides the proportion of agreement among respondents. Consequently, the indicator of discrepancy for a given period in time can be formalised as:

2 $$D_{t} = 1 - \left[ {\frac{{\sqrt {\left( {P_{t} - {\raise0.7ex\hbox{$1$} \!\mathord{\left/ {\vphantom {1 3}}\right.\kern-\nulldelimiterspace} \!\lower0.7ex\hbox{$3$}}} \right)^{2} + \left( {E_{t} - {\raise0.7ex\hbox{$1$} \!\mathord{\left/ {\vphantom {1 3}}\right.\kern-\nulldelimiterspace} \!\lower0.7ex\hbox{$3$}}} \right)^{2} + \left( {M_{t} - {\raise0.7ex\hbox{$1$} \!\mathord{\left/ {\vphantom {1 3}}\right.\kern-\nulldelimiterspace} \!\lower0.7ex\hbox{$3$}}} \right)^{2} } }}{{\sqrt {{\raise0.7ex\hbox{$2$} \!\mathord{\left/ {\vphantom {2 3}}\right.\kern-\nulldelimiterspace} \!\lower0.7ex\hbox{$3$}}} }}} \right].$$

This metric is bounded between zero and one, and conveys a geometric interpretation. The centre of the simplex corresponds to the point of maximum disagreement, indicating that the answers are equidistributed among the three response categories. Conversely, each of the $N$ vertexes corresponds to a point of minimum disagreement, where one category draws all the answers and $D_{t}$ reaches the value of zero.

**Fig. 2:**
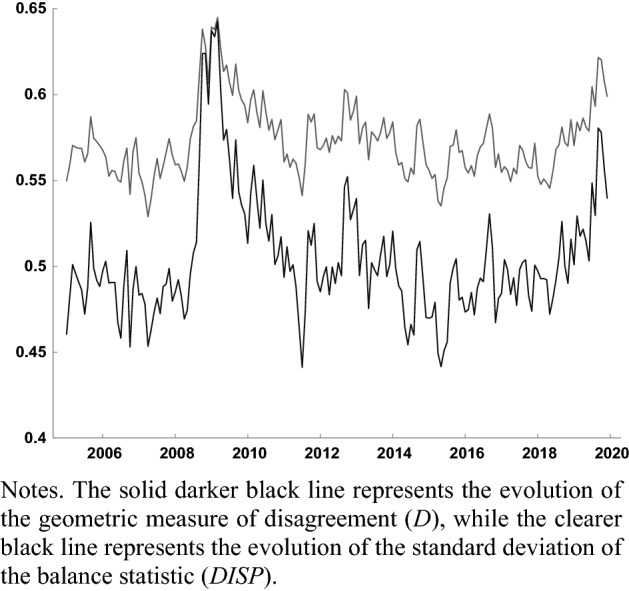
Evolution of disagreement measures for firms’ expectations about industrial production in the EA (2005.01–2019.12)

**Fig. 3:**
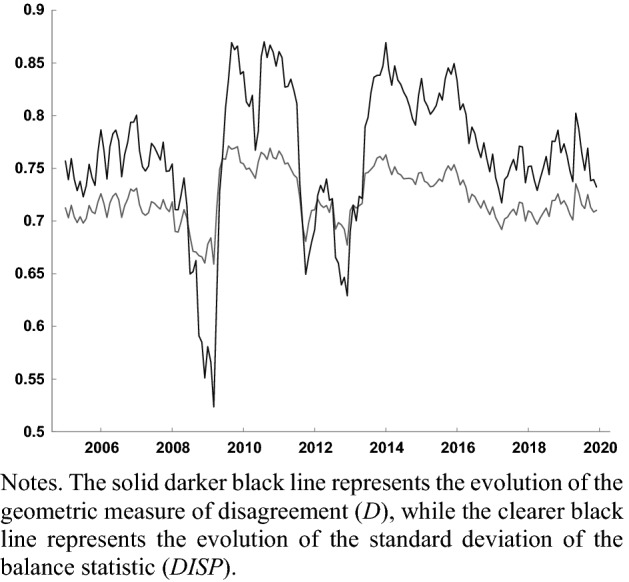
Evolution of disagreement measures for households’ expectations about the general economic situation in the EA (2005.01–2019.12)

Figures 2 and 3 compare the evolution of the geometric measure of disagreement (2) to that of the standard deviation of the balance (1) in the EA, both for the question regarding firms’ expectations about future production (Fig. 2), and for households’ expectations about the general economic situation (Fig. 3). In both cases the metrics of disagreement co-evolve. The correlation between *D* and DISP regarding expectations about production is 0.955, while the correlation between both indicators for consumer expectations regarding the general economic situation is 0.904. Claveria (2021a) obtained a high positive correlation between measures (1) and (2) of disagreement, and found that the main difference between both measures mainly lied in their average level and dispersion, being DISP more volatile and higher in most countries. By means of a simulation experiment, Claveria et al. (2019) showed that the omission of neutral responses in (1) resulted in an overestimation of the level of disagreement.

In this study, expression (2) is used to measure discrepancy in manufacturing surveys (DB) and in consumer surveys (DC). Table 1 contains the summary statistics of disagreement in business and consumer surveys.

**Table 1:** Descriptive and correlation analysis—disagreement and GDP growth

	DB		DC	
	Mean	SD	Mean	SD
Austria	0.503	0.059	0.785	0.085
Belgium	0.481	0.073	0.807	0.089
Finland	0.592	0.082	0.741	0.091
France	0.639	0.052	0.693	0.095
Germany	0.447	0.058	0.627	0.085
Greece	0.627	0.066	0.506	0.187
Italy	0.475	0.062	0.755	0.091
Netherlands	0.472	0.055	0.741	0.121
Portugal	0.385	0.087	0.650	0.171
Spain	0.433	0.053	0.747	0.128
United Kingdom	0.679	0.071	0.762	0.116
Euro Area	0.504	0.036	0.764	0.065

	Correlation—GDP growth and DB		Correlation—GDP growth and DC	
	Pearson	Spearman	Pearson	Spearman
Austria	− 0.280 (0.000)	− 0.057 (0.447)	0.279 (0.000)	0.139 (0.062)
Belgium	− 0.544 (0.000)	− 0.478 (0.000)	− 0.219 (0.003)	− 0.306 (0.000)
Finland	− 0.134 (0.073)	− 0.131 (0.079)	− 0.343 (0.000)	− 0.423 (0.000)
France	− 0.410 (0.000)	− 0.329 (0.000)	0.410 (0.000)	0.402 (0.000)
Germany	− 0.441 (0.000)	− 0.274 (0.000)	0.435 (0.000)	0.338 (0.000)
Greece	− 0.323 (0.000)	− 0.373 (0.000)	0.584 (0.000)	0.606 (0.000)
Italy	− 0.433 (0.000)	− 0.228 (0.002)	0.078 (0.298)	− 0.032 (0.667)
Netherlands	− 0.685 (0.000)	− 0.616 (0.000)	0.022 (0.769)	− 0.080 (0.286)
Portugal	− 0.753 (0.000)	− 0.786 (0.000)	0.686 (0.000)	0.453 (0.000)
Spain	− 0.455 (0.000)	− 0.363 (0.447)	0.027 (0.723)	− 0.144 (0.054)
United Kingdom	− 0.007 (0.928)	− 0.033 (0.663)	0.345 (0.000)	0.612 (0.000)
Euro Area	− 0.702 (0.000)	− 0.445 (0.000)	0.500 (0.000)	0.302 (0.000)

Notes: SD denotes standard deviation. DB refers to aggregate disagreement for businesses and DC to aggregate disagreement for consumers. Between brackets, two-tailed *p* value under the null hypothesis of no correlation

**Fig. 4:**
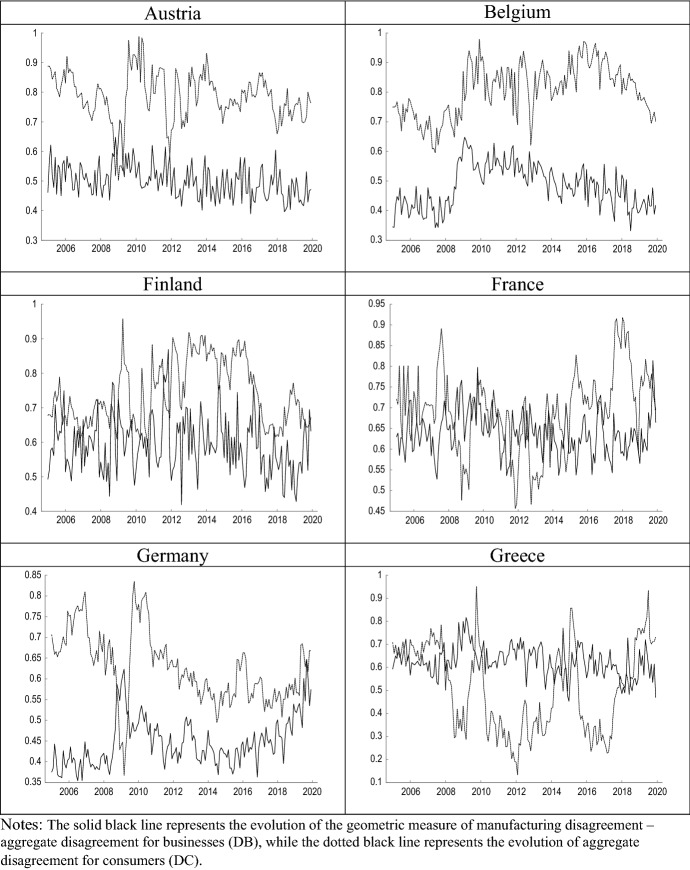

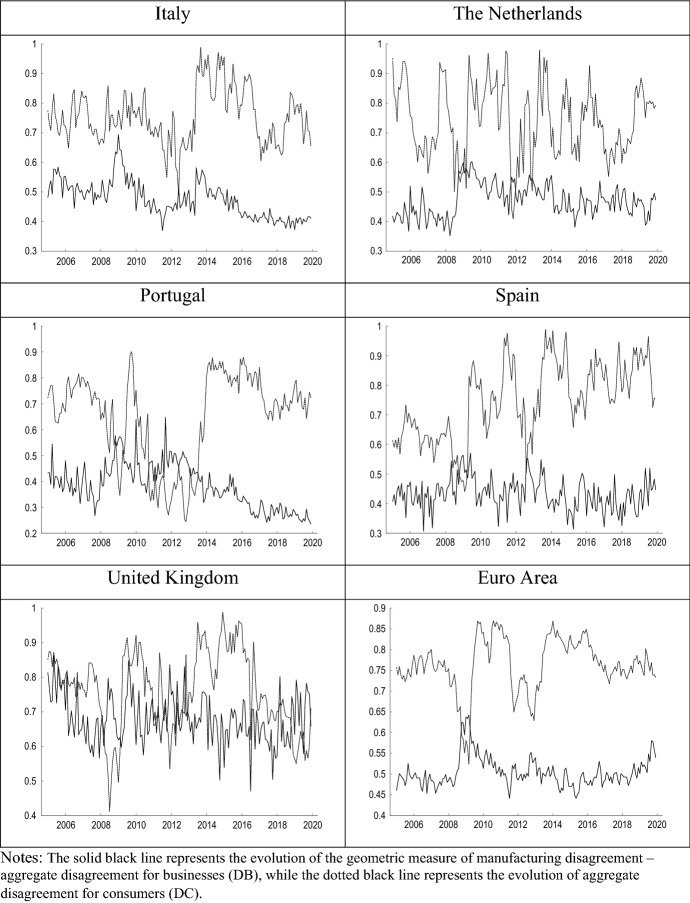
Business disagreement vs consumer disagreement (2005.01–2019.12)

For all countries except Greece, the average degree of DC is higher than DB. This result may have to do both with the differences in the nature of the survey questions and with the fact that the heterogeneity between the panel of households is probably greater than that which may exist among manufacturing companies in a given sector. It should also be noted that in some countries there are remarkable differences between DB and DC. In this sense, Portugal shows the lowest average DB level and Belgium the highest average DC level. Regarding the correlation of disagreement with GDP growth, DB shows a negative correlation in all cases, while DC shows positive correlations with economic growth in all countries except Belgium and Finland. Portugal is the country for which we obtain the highest correlations between disagreement and economic growth dynamics, both for firms and consumers. Finally, Fig. 4 compares the evolution of DB to that of DC in each country, highlighting the negative relationship between both measures in most cases.

To some extent, the observed discrepancies between firms and consumers could be partly attributable to differences in the questions in both surveys: while consumer survey questions refer to objective variables, business surveys questions refer to firm-specific factors. Again, the fact that the heterogeneity between households may be higher than among manufacturing companies, may be also explaining part of these results.

## Empirical results

There exists empirical evidence on the bidirectional relationship between uncertainty and macroeconomic variables (Alessandri and Mumtaz 2019; Caldara et al. 2016; Gilchrist et al. 2014; Glocker and Hölzl 2021; Gupta et al. 2019; Mumtaz and Musso 2021). By means of a VAR approach, in this section, we first examine the dynamic relationship of the discrepancy measures computed in the previous section to gauge the perception of uncertainty and the corresponding macromagnitudes. Independent vector autoregressions are estimated for each country, so no spillover effects are considered. The index $i = 1, \ldots ,N$ denotes the *N* countries analysed in the study. The following bivariate model is used:

3 $$x_{it} = \sum\limits_{p = 1}^{P} {A_{ip} x_{it - p} + \varepsilon_{it} } ,\varepsilon_{it} \sim N\left( {0,\Sigma_{i} } \right).$$

With $x_{it} = \left( {D_{ \cdot ,it} ,z_{it} } \right)$, where $D_{ \cdot } ,_{it}$ refers to the proposed disagreement measure for businesses (DB) and consumers (DC), respectively, and, $z_{it}$ refers to the macroeconomic variable of reference, which in our case is output growth for the *i*-th country at time *t* $\left( {t = 1, \ldots ,T} \right)$. The number of lags, *p*, is selected by means of Schwarz’s Bayesian information criterion (BIC). Heteroscedasticity-consistent standard errors are used for the estimation. A Cholesky decomposition of the covariance matrix is used, ordering the uncertainty proxies first (Bloom 2009). Thus, in the resulting two-variable VAR models, each of the uncertainty measures (DB and DC) is related to GDP growth.

In order to test the robustness of the results, the empirical analysis is replicated in the Appendix using quarterly frequencies. To this end, monthly survey expectations are averaged for each quarter. Additionally, given that the consumer survey has a non-response option, the results for households are compared to those obtained using an alternative criterion for the construction of the geometric indicator of discrepancy, in which the proportion of non-response is equalised between the different categories instead of adding it with the no-change option.

**Fig. 5:**
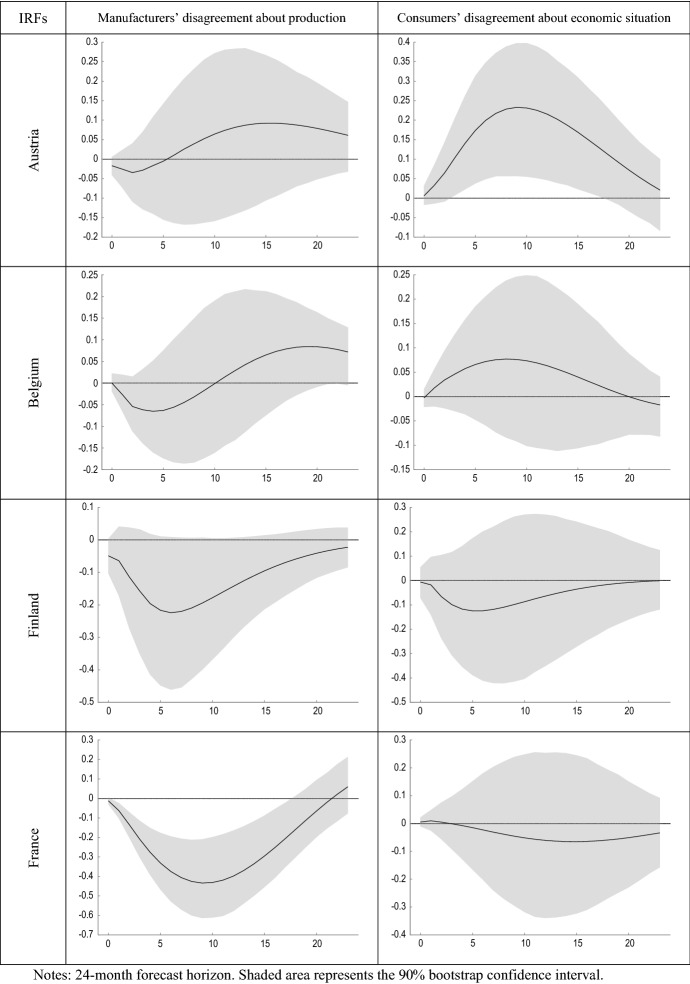

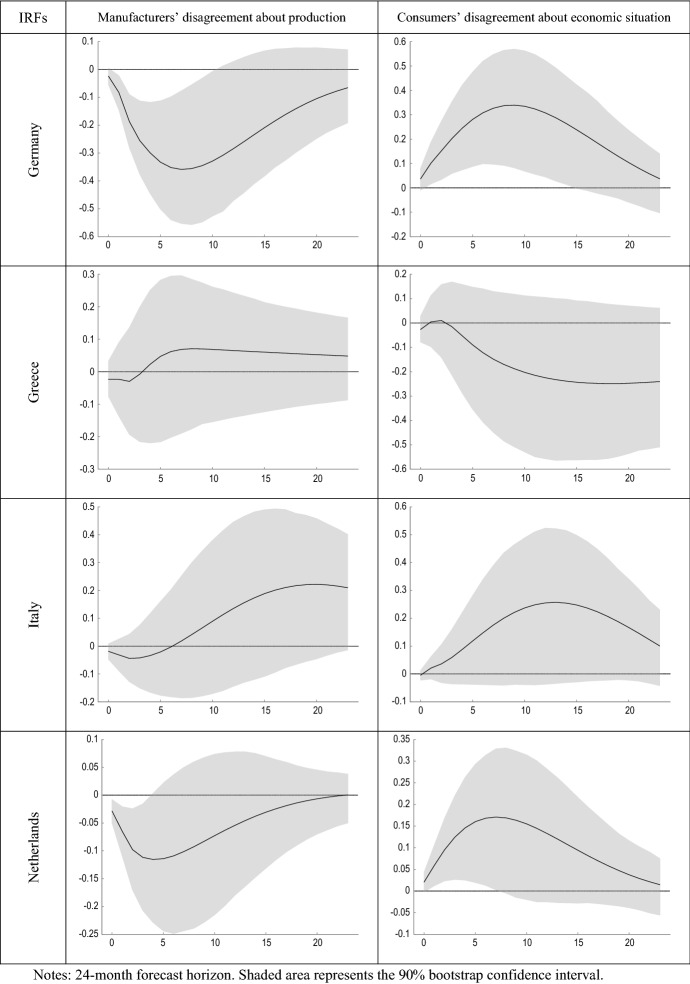

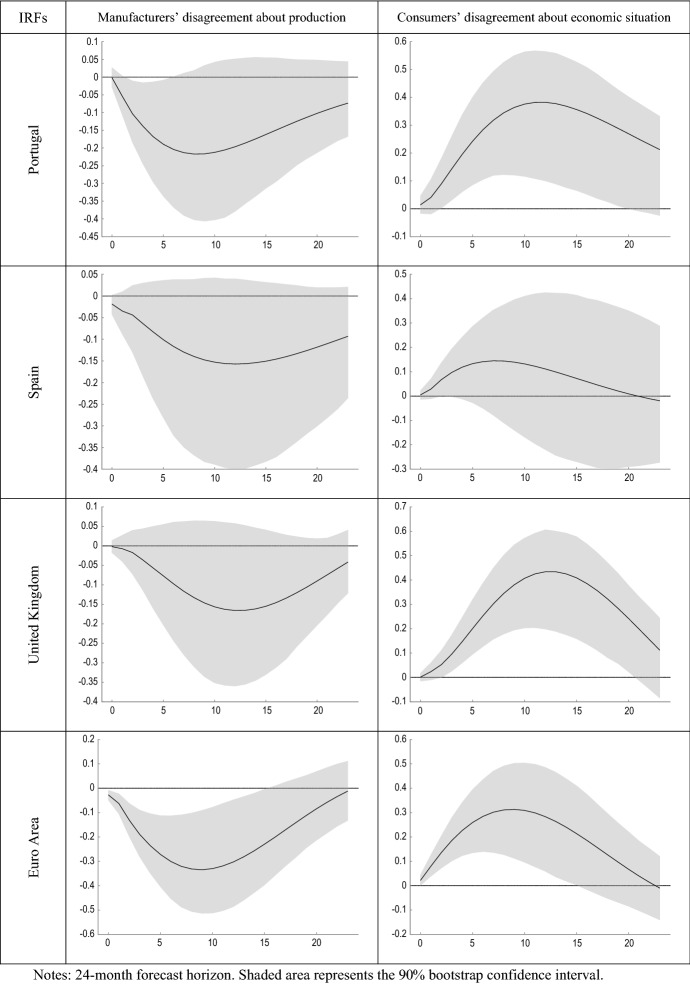
IRFs of GDP growth to shocks in disagreement

**Fig. 6:**
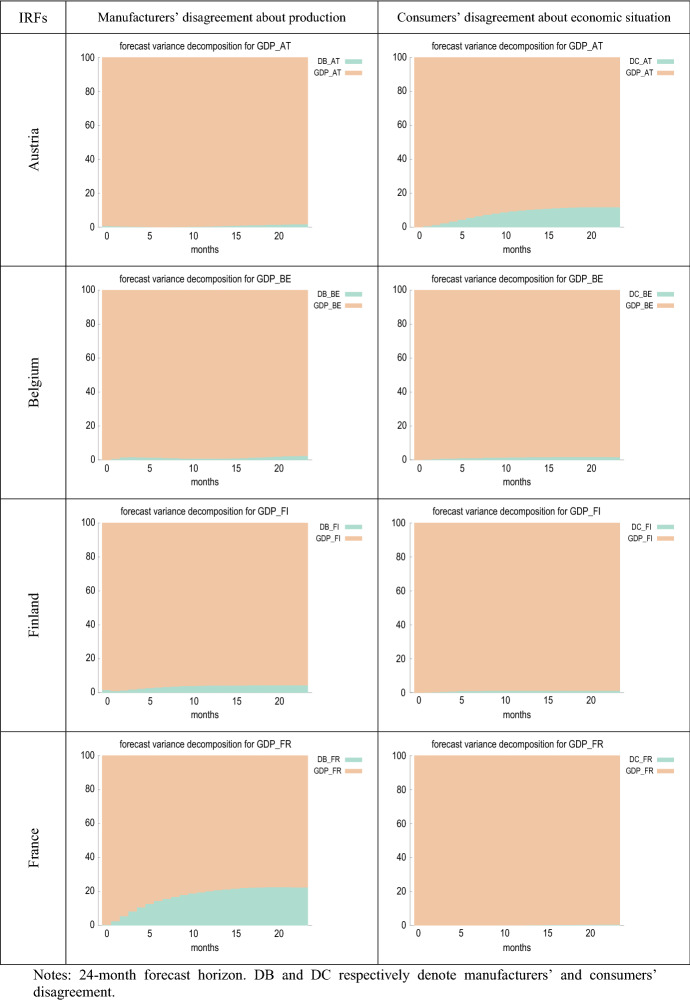

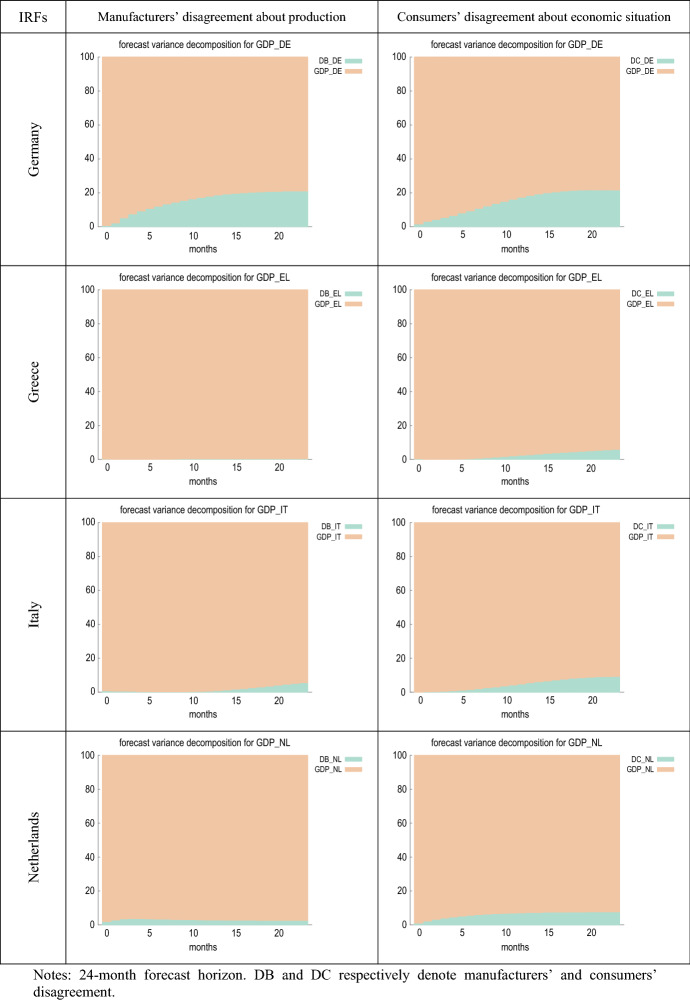

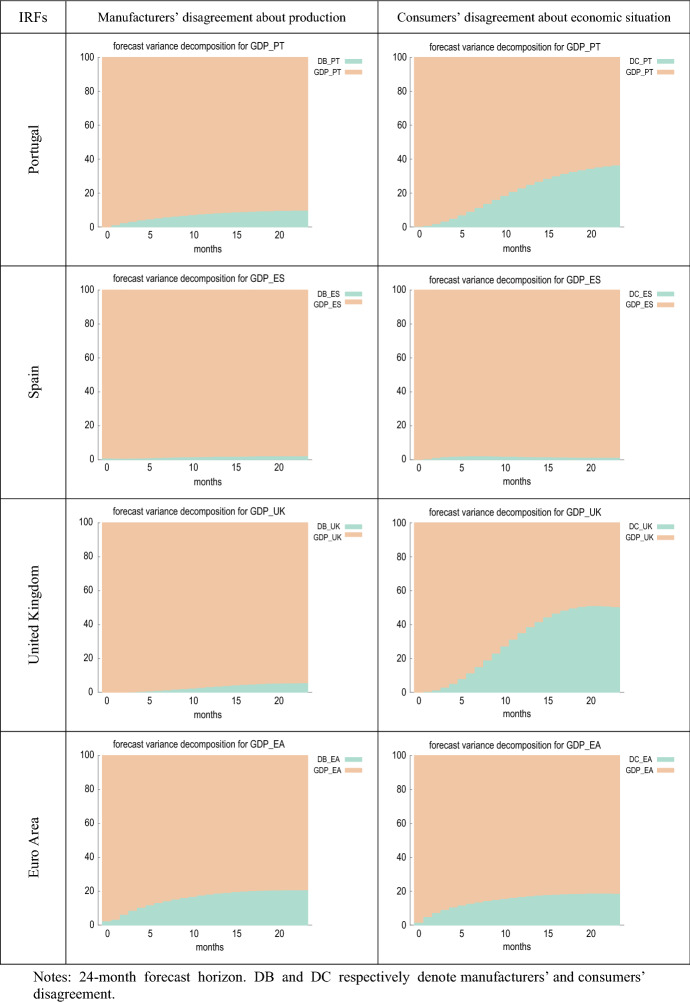
FEVDs for GDP growth (% change over same month in previous year)

Figure 5 compares the estimated impulse response functions (IRFs) of output growth to innovations in manufacturers’ and consumers’ perception of uncertainty. Figure 6 presents the forecast error variance decomposition (FEVD) of GDP growth, which provides information about the relative importance of each innovation in affecting the forecast error variance.

Figure 6 shows that, in most cases, the fraction of the forecast error variance of GDP growth can be mainly attributed to orthogonalised shocks to itself. However, in France, Germany and the EA, the contribution of innovations in firms’ disagreement (DB) is about 20%. A similar result is found for Austria, Germany, Portugal, the UK and the EA in the case of consumer disagreement (DC), where the proportion attributable to innovations in DC is higher than in the rest of the countries, especially in the UK, where it even exceeds 40%.

Figure 5 shows that shocks in DB mostly have a negative effect on economic growth. This result is in line with previous research (Alexopoulos and Cohen 2015; Cerda et al. 2018; Charles et al. 2018; Istiak and Serletis 2018; Meinen and Roehe 2017). Rossi and Sekhposyan (2017) found similarity in the uncertainty cycles across the EA, with some evidence of divergence after the last recession. Jo and Sekkel (2019) found that uncertainty had a persistent negative impact on real economic activity in the US. Recently, Altig et al. (2020) considered several economic uncertainty indicators for the US and UK before and during the COVID-19 pandemic, and found that all indicators showed huge uncertainty jumps in reaction to the pandemic and its economic fallout, but that peak amplitudes differed greatly between the different proxies. Similarly, using historical forecasting errors, Reifschneider and Tulip (2019) found evidence that estimates of uncertainty about future real activity in the US increased after the financial crisis.

While the evidence found in relation to the effect that business uncertainty has on output growth is in line with economic theory and with previous literature, the results obtained for consumers in many cases have the opposite sign. For example, Sahinoz and Cosar (2020) recently found that Turkish firms’ and consumers’ uncertainties were positively correlated. The possible reasons for this finding, partly contrary to what might be expected, may be due to different factors. On the other hand, it should be noted that the uncertainty measure used in the study is based on an indicator of geometric discrepancy, and therefore is based on the disagreement between the agents. As pointed out by Pellegrino (2021), the fact that uncertainty measures are not fully embedded in the econometric models at the estimation stage might cause measurement errors in the regressors and lead to an endogeneity bias. Consequently, the results that have been obtained can also be partially explained by the use of indicators of disagreement as a proxy for economic uncertainty.

On the other hand, another reason for this finding is the different nature of the questions between business and consumer surveys, in the sense that manufacturers’ expectations refer to firm-specific factors, while consumers’ expectations refer to the general economic situation. This, together with the greater heterogeneity that could be expected in the sample of households compared to that of manufacturing firms in the same sector, could in turn explain that the average degree of consumer disagreement was found to be greater than that of firms and evolved in an inverse way (Table 1). As showed by Lahiri and Sheng (2010), aggregate forecast uncertainty can be expressed as the disagreement among the forecasters plus the perceived variability of future aggregate shocks. Therefore, it could be the case that this later component of forecast uncertainty, namely the expected variability of aggregate shocks, is much higher between consumers. Consequently, the inherent difference in the composition of both groups of respondents could be explaining the different results found regarding the effect that unexpected increases in the disagreement of both types of agents have on the volatility of the growth of economic activity.

Finally, as mentioned above, the analysis carried out focuses fundamentally on the comparison between both types of agents (firms and consumers), and does not take into account country spillovers and additional variables. As pointed out by Carriero et al. (2018), potential biases may arise from the omission of variables due to restricted information sets in country-specific analysis. The use of panel local projections (Jordà 2005; Plagborg-Møller and Wolf 2021) could be one way to circumvent this issue. In a recent paper, Caggiano and Castelnuovo (2021) used a dynamic hierarchical factor model to disentangle the global component form the country-specific ones. Therefore, it should also be noted that some of the obtained results may be conditioned by the setup of the analysis.

## Conclusion

This study analyses the effect on economic growth of shocks in the perception of uncertainty of firms and consumers. We use qualitative survey data about the expected direction of change in production and in economic activity to proxy economic uncertainty, both form the supply and the demand sides of the economy. Agents’ perception of uncertainty is gauged by a geometric indicator of discrepancy in survey expectations to construct aggregate disagreement indicators for both firms and consumers. First, when comparing the level of disagreement between business and consumer surveys in eleven European countries and the Euro Area, it is found that the average degree of consumer disagreement is greater than that of manufacturers, which could be due in part to the greater heterogeneity that might be expected between the former and the different nature of the questions in both surveys.

Second, the dynamic relationship between innovations in perceived economic uncertainty and economic growth is assessed by estimating the impulse response functions using a vector autoregressive framework. The obtained results differ markedly between disagreement in business and in consumer surveys. On the one hand, shocks to consumer discrepancy are generally found to be of greater magnitude and duration than those to manufacturer discrepancy. On the other hand, while shocks to business discrepancy lead to a decrease in economic activity, shocks to consumer economic discrepancy tend to have the opposite effect. This finding suggests that the effect of shocks to agents’ perception of uncertainty on economic aggregates would depend on the type of agent and the way in which this perception has been elicited.

Finally, we want to note some of the limitations of the present study. On the one hand, it should be highlighted that the findings of this research may be conditioned by several biases derived from the exogenous measurement of uncertainty and the omission of variables. On the other hand, we want to point out the differences in the nature of the questions between business and consumer surveys, in the sense that firms’ expectations refer to specific factors of the company, while consumers’ expectations refer to the general development of economic activity. Regarding future lines of research, the application of panel local projections to control for time-invariant factors and potential spillovers across countries, as well as the use of nonlinear VAR models to test for the presence of nonlinearities in uncertainty are aspects left for further research. Other aspects to explore are the extension of the analysis to other variables included in the surveys, such as order-book levels, exports or savings, as well as to other surveys.

## Supplementary Information

Below is the link to the electronic supplementary material.

Supplementary file1 (DOCX 183 kb)

## Data Availability

The datasets used and/or analysed during the current study are: The *Joint Harmonised EU Consumer Survey* conducted by the European Commission, which can be freely downloaded at: https://ec.europa.eu/info/business-economy-euro/indicators-statistics/economic-databases/business-and-consumer-surveys_en. Gross Domestic Product (GDP) provided by Eurostat: http://ec.europa.eu/eurostat/web/lfs/data/database.
