# Supplementary material for: Disagreement on expectations: firms versus consumers
Source: SN Bus Econ. 2022 Jan 10;1(12):161. doi: 10.1007/s43546-021-00164-4 (PMC8744376; doi:10.1007/s43546-021-00164-4)
Supplement: Supplementary file 1 — Supplementary file1 (DOCX 183 kb) [file 43546_2021_164_MOESM1_ESM.docx]

**Appendix**

In order to test the robustness of the obtained results, the empirical analysis is replicated using (a) quarterly frequencies and, (b) an alternative design of the geometric indicator of discrepancy. To check whether results hold when using the original GDP growth series, i.e. year-on-year growth rates of quarterly GDP, disagreement measures were averaged for each quarter. Fig. 7 contains the estimated impulse response functions (IRFs) of output growth to innovations in manufacturers’ and consumers’ perception of uncertainty as captured by the quarterly discrepancy measures. With very few exceptions, the obtained results for quarterly data show similar dynamics to those obtained for monthly data.

Additionally, since the consumer survey includes an additional non-response option (‘don’t know’), denoted as N, to check whether the results hold to an alternative design of the geometric indicator of discrepancy, DC was computed using an alternative aggregating scheme. The new measure of consumer disagreement, denoted as DC*, was calculated so that the share of non-responses is equalised between the different categories rather than aggregated with the no-change proportion (E). Fig. 8 shows the estimated impulse response functions (IRFs) of GDP growth to innovations in consumers’ perception of uncertainty as captured by DC*. Table 2 contains the summary statistics for DC*. It can be observed that the impact of including the share of ‘don’t know’ answers in the ‘no-change’ category when computing the disagreement indicator is almost imperceptible. The main reason for this finding lies in the fact that few responses fall within the N category in the consumer survey carried out by the European Commission.

**Fig. 7.** IRFs of quarterly GDP to shocks in disagreement

| IRFs | Manufacturers’ disagreement about production | Consumers’ disagreement about economic situation |
| --- | --- | --- |
| Austria |  |  |
| Belgium |  |  |
| Finland |  |  |
| France |  |  |

Notes: 12-quarter forecast horizon. Shaded area represents the 90% bootstrap confidence interval.

**Fig. 7** (cont.1)**.** IRFs of quarterly GDP to shocks in disagreement

| IRFs | Manufacturers’ disagreement about production | Consumers’ disagreement about economic situation |
| --- | --- | --- |
| Germany |  |  |
| Greece |  |  |
| Italy |  |  |
| Netherlands |  |  |

Notes: 12-quarter forecast horizon. Shaded area represents the 90% bootstrap confidence interval.

**Fig. 7** (cont.2)**.** IRFs of quarterly GDP to shocks in disagreement

| IRFs | Manufacturers’ disagreement about production | Consumers’ disagreement about economic situation |
| --- | --- | --- |
| Portugal |  |  |
| Spain |  |  |
| United Kingdom |  |  |
| Euro Area |  |  |

Notes: 12-quarter forecast horizon. Shaded area represents the 90% bootstrap confidence interval.

**Fig. 8.** IRFs of GDP to shocks in consumers’ disagreement about the economic situation

| IRFs | DC | DC* |
| --- | --- | --- |
| Austria |  |  |
| Belgium |  |  |
| Finland |  |  |
| France |  |  |

Notes: 24-month forecast horizon. Shaded area represents the 90% bootstrap confidence interval.

**Fig. 8** (cont.1)**.** IRFs of GDP to shocks in consumers’ disagreement about economic situation

| IRFs | DC | DC* |
| --- | --- | --- |
| Germany |  |  |
| Greece |  |  |
| Italy |  |  |
| Netherlands |  |  |

Notes: 24-month forecast horizon. Shaded area represents the 90% bootstrap confidence interval.

**Fig. 8** (cont.2)**.** IRFs of GDP to shocks in consumers’ disagreement about economic situation

| IRFs | DC | DC* |
| --- | --- | --- |
| Portugal |  |  |
| Spain |  |  |
| United Kingdom |  |  |
| Euro Area |  |  |

Notes: 24-month forecast horizon. Shaded area represents the 90% bootstrap confidence interval.

**Table 2**

Summary statistics (2005.01-2019.12)

|  | DC* | |
| --- | --- | --- |
|  | Mean | SD |
| Austria | 0.794 | 0.086 |
| Belgium | 0.840 | 0.074 |
| Finland | 0.751 | 0.091 |
| France | 0.717 | 0.111 |
| Germany | 0.675 | 0.081 |
| Greece | 0.490 | 0.193 |
| Italy | 0.772 | 0.089 |
| Netherlands | 0.742 | 0.123 |
| Portugal | 0.661 | 0.181 |
| Spain | 0.768 | 0.121 |
| United Kingdom | 0.751 | 0.118 |
| Euro Area | 0.798 | 0.076 |
